# Signaling beyond Punching Holes: Modulation of Cellular Responses by *Vibrio cholerae* Cytolysin

**DOI:** 10.3390/toxins7083344

**Published:** 2015-08-21

**Authors:** Barkha Khilwani, Kausik Chattopadhyay

**Affiliations:** Centre for Protein Science, Design and Engineering, Department of Biological Sciences Indian Institute of Science Education and Research Mohali Sector 81, S. A. S. Nagar, Manauli PO 140306, Punjab, India; E-Mail: barkhak.515@gmail.com

**Keywords:** *Vibrio cholerae* cytolysin, pore-forming toxin, cytotoxin, membrane, cell signaling

## Abstract

Pore-forming toxins (PFTs) are a distinct class of membrane-damaging cytolytic proteins that contribute significantly towards the virulence processes employed by various pathogenic bacteria. *Vibrio cholerae* cytolysin (VCC) is a prominent member of the beta-barrel PFT (beta-PFT) family. It is secreted by most of the pathogenic strains of the intestinal pathogen *V. cholerae*. Owing to its potent membrane-damaging cell-killing activity, VCC is believed to play critical roles in *V. cholerae* pathogenesis, particularly in those strains that lack the cholera toxin. Large numbers of studies have explored the mechanistic basis of the cell-killing activity of VCC. Consistent with the beta-PFT mode of action, VCC has been shown to act on the target cells by forming transmembrane oligomeric beta-barrel pores, thereby leading to permeabilization of the target cell membranes. Apart from the pore-formation-induced direct cell-killing action, VCC exhibits the potential to initiate a plethora of signal transduction pathways that may lead to apoptosis, or may act to enhance the cell survival/activation responses, depending on the type of target cells. In this review, we will present a concise view of our current understanding regarding the multiple aspects of these cellular responses, and their underlying signaling mechanisms, evoked by VCC.

## 1. Introduction

Cholera is a deadly diarrhoeal disease caused by the gram negative bacterium *Vibrio cholerae*. The major virulence factor responsible for the induction of the pathophysiological responses during *V. cholerae* infection is cholera toxin that is encoded by the CTX bacteriophage (CTXФ) [1]. However, infections with *V. cholerae* strains lacking the cholera toxin has also been found to cause cholera-like symptoms suggesting the implications of the additional virulence factors for the disease development [2,3].

Many pathogenic strains of *V. cholerae* secrete a cytolysin/cytotoxin known as *Vibrio cholerae* cytolysin (VCC) [2,4,5]. VCC was initially identified as a protein factor secreted by pathogenic strains of *V. cholerae* that causes hemolysis of sheep erythrocytes [6]. Subsequently, VCC has been shown to exhibit potent cell-killing activity against a wide array of target eukaryotic cells [5]. Experiments using animal models of cholera have also demonstrated prominent enterotoxic property of VCC [7]. Based on these observations, VCC is considered as a potential virulence factor of *V. cholerae*. However, the exact role of VCC in the *V. cholerae* pathogenesis process still remains obscure.

In its mode of action, VCC belongs to the family of β-barrel pore-forming toxins (β-PFTs) [8,9,10,11,12]. Pore-forming toxins (PFTs) are a unique class of protein toxins that damage the host cell membranes by forming transmembrane pores [13]. PFTs are classified as α-PFTs and β-PFTs [14,15] based on the structural motif involved in the membrane pore-formation. β-PFTs, characterized by the formation of β-barrel structures in the target membranes, include the majority of the bacterial PFTs that cause membrane damage and play important roles in the virulence mechanisms of those bacteria [16]. Some typical examples of β-PFTs include aerolysin from *Aeromonas hydrophila* and α-hemolysin from *Staphylococcus aureus* [17,18]. Bacterial β-PFTs are, in general, secreted as soluble monomeric proteins, and in contact with the target cell membrane they form transmembrane oligomeric pores [19]. This ability of the β-PFTs to punch holes in membrane enables them to damage the integrity of the cellular architecture. However, apart from the pore-forming action, β-PFTs can also activate a plethora of cellular responses depending on the host cell type, and the dosage of the toxin [20]. A cell, upon attack by β-PFTs, initiates multiple signaling cascades that may either lead to apoptotic death of the target cells or may trigger repair mechanisms [16].

As mentioned above, based on the overall structural organization and mode of action, VCC has been characterized as a prototype member in the β-PFT family [21]. Studies done with a wide array of cell types have shown that VCC causes cell death by forming transmembrane oligomeric β-barrel pores [22,23]. Formation of such transmembrane pores causes cell killing either via generation of colloid-osmotic imbalance in the target cells [24], or via induction of apoptosis in a caspase-dependent manner [3]. Sub-lytic concentrations of VCC, on the other hand, have been shown to modulate the cellular machinery in such a way that the target cells are activated to trigger pathways for promoting cell survival [25,26,27]. Such diversity of responses induced by VCC suggests a significant yet unidentified role of this atypical β-PFT in the virulence mechanism of *V. cholerae*. Nevertheless, it is now well-established, that VCC may act as a significant accessory toxin in *V. cholerae*, particularly in those strains that lack the gene for cholera toxin. The present review attempts to summarize various studies undertaken to understand the cellular responses evoked by VCC.

## 2. Structural Features of VCC

VCC is encoded by the *hlyA* gene [28], in the form of a precursor protein, pre-pro-VCC of molecular mass ~82 kDa [4]. This precursor molecule undergoes two-step processing to generate the mature form of the toxin having a molecular mass of ~65 kDa [29]. During secretion through the bacterial inner membrane, the N-terminal leader sequence is cleaved, thus releasing an inactive precursor form, Pro-VCC, into the extracellular space [4,29]. Subsequently, proteolytic removal of an *N*-terminal Pro-domain generates the active mature form of VCC [10,30].

**Figure 1:**
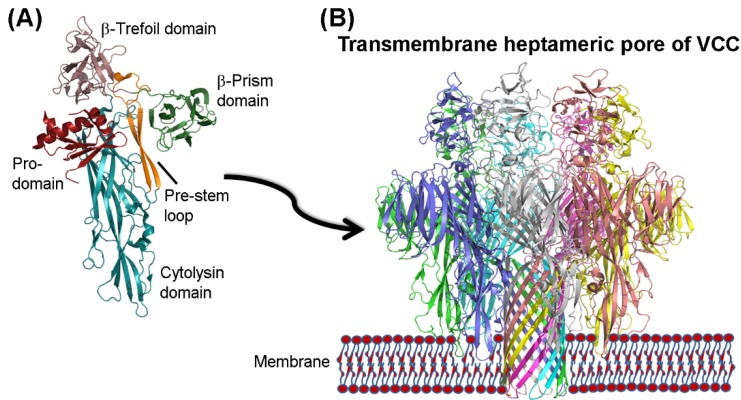

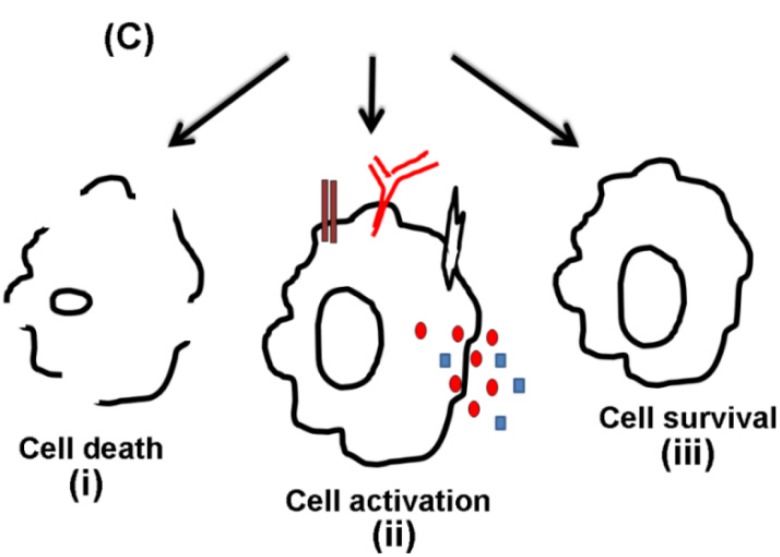
(**A**) Crystal structure of VCC highlights multiple domains and structural motifs (Protein data bank (PDB) entry: 1XEZ); (**B**) Transmembrane heptameric pore of VCC (PDB entry: 3O44). Structural models are visualized using the program PyMOL (DeLano WL, The PyMOL Molecular Graphics System 2002); (**C**) Schematic representation of the selected cellular responses initiated by VCC. (i) VCC-mediated membrane pore-formation can cause cell killing via colloid-osmotic lysis, or by triggering apoptosis; (ii) VCC can trigger activation of the target cells resulting into an array of cellular responses; (iii) Activation of target cells may aid in the process of toxin clearing and cell survival.

High-resolution three-dimensional structure has been determined for Pro-VCC [10]. The structural model shows a cross-shaped structure divided into four structural domains with distinct functionalities [10] (Figure 1A). The N-terminal pro-domain possesses chaperon-like activity [30] and is important for the proper secretion and folding of the toxin molecule [31]. The central scaffold of the VCC toxin is the cytolysin domain, and it shares structural homology to core cytolysin domains of the archetypical β-PFTs, that include *S. aureus* α-hemolysin and staphylococcal LukF [9]. The cytolysin domain of VCC harbours the structural motif(s) that recognize membrane lipid components, thus playing a critical role in mediating the membrane-binding step of the toxin [32,33]. The cytolysin domain also contributes towards the structural motif, the pre-stem loop, which participates in the formation of the transmembrane β-barrel segment during the membrane pore-formation process [34]. VCC appears to be an atypical β-PFT based on the presence of two accessory lectin-like domains at the *C*-terminal boundary of the cytolysin domain: a β-trefoil and a β-prism lectin-like domain [10,21]. In particular, the β-prism lectin-like domain acts as the structural scaffold for a specific lectin-like activity toward β1-galactosyl-terminated glycoconjugates [35]. Such an activity of the β-prism domain aids in the binding of the toxin to the glycan receptors present on the target cells. Moreover, β-prism domain’s lectin activity appears to act as a regulatory switch to control the membrane pore-formation mechanism of VCC [21,35].

## 3. Mechanism of Membrane Pore-Formation

Consistent with the β-PFT mode of action, membrane pore-formation mechanism of VCC involves following sequence of events. Water-soluble monomers of the toxin bind to the target cell membranes, and then assemble into transient pre-pore oligomeric intermediates. Subsequently, the pore-forming pre-stem loops from each of the toxin protomers insert into the membrane in a concerted manner to generate the transmembrane oligomeric β-barrel pores [11,21,36] (Figure 1B). The pores formed by VCC are anion-selective with an internal diameter of 1–2 nm [23,37].

Membrane pore-formation mechanism of VCC is regulated by multiple factors. For example, the membrane-binding step is facilitated by a complex cross talk between VCC and membrane components. Amphipathicity-driven partitioning of VCC into the membrane lipid bilayer is believed to trigger non-specific association of the toxin with the target membranes [38]. However, specific interaction of VCC with the membrane lipid head-groups appears to represent a key triggering factor to drive the efficacy of membrane binding and subsequent pore-formation process [8,39]. The presence of cholesterol and sphingolipids also plays critical roles in regulating the pore-formation mechanism of VCC [40,41]. In addition, specific interaction of VCC with the glycan receptors present in the target cell membranes has been implicated for the membrane binding and pore-formation process [35]. However, the identity of any specific receptor for VCC has not been determined yet. Apart from the membrane binding step, subsequent oligomerization and membrane insertions events also depend critically on multiple regulatory factors, most of which are related to the efficacy of the interactions made by VCC with the membrane components.

## 4. Cytotoxic Effects

PFTs play significant roles in the virulence mechanisms of various pathogenic bacteria [13]. Membrane pores formed by these toxins aid in the virulence processes, either by allowing bacterial components to enter into the target cells through the membrane pores, or by inducing colloid-osmotic lysis of the cells. VCC permeabilizes the host cell plasma membranes and disturbs the osmotic balance within the target cells leading to the cell lysis (Figure 1C). VCC has been initially identified as a hemolysin protein produced by the non-O1 strains of *V. cholera* [2]. VCC causes lysis of erythrocytes from various species [4,5]. Nucleated cells are more resistant to the attack by VCC, but at higher concentrations of the toxin, cell lysis has been observed in the epithelial cells and macrophages [4,5]. Apart from the colloid-osmotic lysis-mediated direct cell-killing, PFTs are known to induce other cytopathic effects that lead towards cell death; for example, necrosis, as in the case of α-toxin of *Clostridium septicum*, staphylococcal Panton-Valentine leukocidin (PVL) [42,43]. A few PFTs have been reported to induce inflammatory cell death known as pyroptosis. Examples include the PFT from *Bacillus anthracis* that causes pyroptosis of macrophages, and SLO that induces cell death by pyroptosis in mouse bone marrow-derived macrophages [44,45]. In the following section, we focus on the mechanisms, apart from the pore-formation-induced direct cell-lysis, that contribute toward the cytotoxic effects generated by VCC (Table 1).

**Table 1:** Cytotoxic responses triggered by VCC.

Cytotoxic effect	Cell Type	Effects	References
Apoptosis	Int 407	Efflux of intracellular K^+^, no intra-nucleosomal degradation, and pores impermeable to Ca^2+^	[5]
	Caco2 and CHO cells	Caspase-3 Activation	[25]
	B1a Cells	Caspase-9/-8-dependent apoptosis, lymphocyte apoptosis	[46]
	Mouse Peritoneal Macrophages	Caspase-9 dependent apoptosis independent of TLR (Toll-like Receptors)	[47]
Vacuolation	HeLa Cells	VCC from clinical strains of the *V. cholerae* induces vacuole formation	[48]
	Vero Cells	VCC present in the culture supernatants of *V. cholerae* Amazonia causes vacuolation Purified VCC also shows vacuolation effect	[49]
	T84 and MDCK-1	High concentrations of VCC causes damage to the epithelium by stimulating vacuole formation	[50]
	Vero cells and BHK cells	Channel formation by VCC is necessary for vacuolating phenotype	[50]

### 4.1. Apoptosis Induced by VCC

Apoptosis is the phenomenon wherein cells undergo a series of events resulting in the programmed cell death. Apoptosis is characterized by nuclear condensation, DNA fragmentation and an overall collapse of the cellular machinery [51]. Bacterial PFTs like listeriolysin O (LLO) cause apoptosis of murine T-lymphocytes *in vivo* and *in vitro* [52]. Panton-Valentine Leukocidine (PVL), a β-PFT from *S. aureus* induces apoptosis in neutrophils [53]. PFT-induced apoptosis can be caspase-dependent as in the case of α-toxin of *S. aureus* which initiates the intrinsic death pathway in the Jurkat cells [54].

VCC has been shown to cause cytolysis of intestinal cells, but there has been no evidence showing the inter-nucleosomal DNA degradation as observed in the case of staphylococcal α-toxin [5,55]. However, in-depth studies by various groups have identified VCC as a toxin leading to apoptosis in a caspase-dependent manner [3,47]. In separate studies, roles of caspase-3 and caspase-9 in VCC-induced apoptosis have been reported [3,46]. An increase in the sub-diploid DNA content in the Caco-2 cells and COS-7 cells has been observed upon VCC treatment. Apoptosis induction by VCC has been further characterized to involve chromatin condensation, nuclear fragmentation and blebbing of the plasma membranes [3]. Recently, it has also been reported that VCC leads to TLR-independent apoptosis in murine peritoneal cavity macrophages [47].

### 4.2. Cell Vacuolating Activity

Vacuole formation is a common phenomenon observed in the cells in response to several organic and inorganic compounds and toxins [56]. *Helicobacter pylori*, a gastric pathogen, secretes vacuolating cytotoxin A (VacA) that causes vacuolation in the various cell types, and plays a significant role in the virulence process of *H. pylori* [57]. Bacterial PFTs, aerolysin from *A. hydrophila* and hemolysin from *Serratia marcences* cause cellular vacuolation [58,59]. It has been reported previously that some of the clinical strains of *V. cholerae* induce cell vacuolation, and the factor responsible for such effect has been identified to be VCC [48,49]. Intracellular vacuoles caused by VCC have been further characterized to be acidic in nature as indicated by uptake of neutral red. It has been determined that the pore formation is a necessary step for vacuole formation by VCC [50]. In the process of cell vacuolation, VCC migrates from the plasma membranes to the early-to-late endosomes, and finally to the lysosomes [50]. It has been reported that VCC induces formation of vacuoles in the intestinal cells of *Caenorhabditis elegans* [60].

## 5. Cell Activation by VCC

Gut pathogens like *V. cholerae* damage the gastro-intestinal track and release virulence factors to establish pathogenesis. The damages caused, in turn, may activate the host immune system and allow recruitment of the immune cells at the site of infection. Initial encounter of the pathogen is made with the cells of the innate immune system, which further may trigger the adaptive immune system. The innate immune system, the first line of defense, consists of various cells that include macrophages, mast cells and dendritic cells. These cells either phagocytose the pathogen and/or the pathogen-derived factors or produce cytokines and chemokines to prevent the spread of the pathogen in the host system [61]. Receptors present on the surface of these cells recognize the pathogen-associated molecular patterns (PAMPs) and generate specific responses. PFTs can be recognized by these Pattern Recognition Receptors (PRRs), like Toll-like receptors (TLRs) and Nod-like receptors (NLRs), and upon stimulation with a particular PFT a specific response can be initiated within the cells. In this part, we will highlight the array of cell activation processes that have been documented in response to VCC (Table 2) (Figure 1C).

**Table 2:** Cell activation responses elicited by VCC.

Cell Type	Effects	References
T84 cells	IL-8-dominates inflammatory response, and also upregulation of IL-6 and TNFα	[27]
B1a cells	TLR2-dependent NFκB activation and upregulation of CD25 surface expression	[46]
Mouse bone marrow derived mast cells	Production of IL-4, IL-5, IL-6 and TNFα and IgE-dependent activation	[62]
RAW 264.7 and THP-1 cells	Initiation of pro-inflammatory signaling cascades in response to transmembrane VCC oligomer	[63]

### 5.1. Association of VCC with the Pattern Recognition Receptors

Recognition of an antigen by the antigen presenting cells (APCs) is one of the first steps in the activation of cellular responses. VCC activates cells of the immune system by binding mainly to the TLR2. In mouse bone marrow-derived mast cells, both the VCC monomer and the oligomer up-regulate surface-expression of TLR2 [62]. Other PFTs are also known to activate TLR-dependent responses. For example, anthrolysin O up-regulates TLR4 expression in mouse bone marrow-derived macrophages that induces apoptosis [64]. Similarly, pneumolysin is also recognized by TLR4 to initiate inflammatory cascades in the mouse macrophages, which further protects the cells from the Pneumococcal infection [65]. VCC induces apoptosis in murine macrophages, but it has been found to do so in a TLR-independent manner [47]. Another study has shown that the transmembrane oligomeric form of VCC activates the cells of monocytic lineage via engaging the TLR2/TLR6-dependent signaling pathway, and thus induce proinflammatory cytokine production in TLR-dependent manner [63]. In such response generation, VCC has been found to initiate the MyD88-dependent signaling cascade downstream of the TLR activation process [63].

Apart from TLRs, an important class of PRRs that play a significant role in regulating immune responses against PFTs are the NLRs that are cytoplasmic proteins. NLRs recognize intracellular antigens and activate the signaling cascades leading to caspase-1 activation, which is involved in the membrane repair mechanisms [66]. VCC has been implicated in recognition of pathogenic *V. cholerae* by NLRP3 [67]. Signaling molecules activated downstream of the NLRP3, and the detailed pathway involved in the process is still remain unexplored.

### 5.2. Cytokine Production

Activation of an immune cell by an antigen leads to the release of various cytokines and chemokines that shape the cellular responses in a particular direction. These cytokines/chemokines are considered to be the markers of a particular response, like IL-6, TNF-α production indicates pro-inflammatory response, and release of IL-4 and IL-10 indicate generation of an anti-inflammatory response [61].

In mouse bone marrow-derived mast cells, VCC induces production of IL-4, IL-5 and IL-6, indicating Th2-biased responses [62]. VCC treatment also enhances TNFα production, and activates these cells in an IgE-dependent manner. In T84 cells, an *in vitro* model for human intestinal epithelium, VCC generates an inflammatory response by up-regulating the mRNA expression of IL-8 and TNFα [27]. The transmembrane oligomeric form of VCC activates the cells of the monocytic lineage, to produce pro-inflammatory cytokines like TNFα, and IL-6, and also nitric oxide production [63].

VCC activates various cell types and thus generates variety of responses, depending upon the concentration of the toxin and the type of the host cells under consideration. Activated cells may trigger certain signaling pathways that help in removal of the toxin from the system, or repair the damages caused, thus enabling the survival of the target cells. In the following sections, we explore the “cell survival” pathways induced by VCC.

## 6. Cell Survival in Response to VCC

Membrane pore-formation by VCC damages the integrity of the cell, and in turn the cells may initiate cellular responses that tend to enhance the cell survival. Such mechanisms would involve initiation of different signaling cascades, and that may result in the phenomenon like autophagy, which can protect the cells against the toxin attack (Table 3) (Figure 1C).

**Table 3:** Cell survival responses triggered by VCC.

Cell Type	Effects	References
Caco2 and CHO	Autophagosome formation and degradation of VCC	[68]
HEK	Increased amount of activated LC3 and internalization of VCC by autophagy	[25]
*C. elegans*	Stabilization of HIF-1 inducing hypoxia pathway that leads to enhanced survival	[69]
HaCaT cells	Phosphorylation of p38 inducing p38 mediated pathway	[70]

### 6.1. Autophagic Response

The cellular machinery includes mechanisms to repair the damage, and recycle the affected organelles within the cell. Such a mechanism is known as autophagy [71]. Membrane permeabilization caused by PFTs might thus also induce autophagy to mend the damage [72]. VCC forms transmembrane pores in the host cell membranes, and such membrane-damaging effect is recognized by the cells. This, in turn, activates the pathways to repair the damage [73]. VCC has been implicated in autophagy, and it is found to be seized by the cells in the lumen of the autophagy vacuoles, after internalization in the autophagosomes [25]. In the cell lines like HEK and CHO, VCC initiates the autophagy, which in turn, increases the survival of these cells [68]. Thus, it appears that the VCC-mediated membrane-permeabilization effect may enable the target cells with an opportunity to combat death, by eliciting a survival strategy like the autophagic response.

### 6.2. p38 Activation

MAP kinase (MAPK) p38 has been known to be involved in signaling during stress responses of a cell [74]. When cells are infected with sub-lytic concentrations of PFTs, the plasma membrane gets permeabilized, but the cells are not lysed. Cells can survive, by activating various signaling pathways, many of which involve increasing the levels of phosphorylated p38 [75,76]. Activated p38 may initiate the Unfolded Protein Responses (UPR) or an inflammatory signaling cascade that enhance the survival chances of the target cells [77]. In the cells treated with VCC, levels of phosphorylated p38 have been found to increase, while the transfer of those cells into the high potassium-containing media attenuates the p38 activation, thus implicating the role(s) of the potassium efflux in the PFT-mediated p38 phosphorylation/activation [70,72]. Other prominent bacterial PFTs including streptolysin O (SLO), pneumolysin, and aerolysin, also initiate similar responses when present in the sub-cytolytic amounts [76,78]. Effect of p38 phospohorylation induced by VCC is not yet studied in-depth. However, interestingly the transmembrane oligomeric form of VCC does not show any involvement of p38 MAPK in the pro-inflammatory response induction process [63].

### 6.3. Hypoxia

While studying the cellular responses initiated by PFTs, the nematode *C. elegans* has been used as an invertebrate host to identify and assess the effect of the toxins [79]. In a study done to identify the virulence factors of *V. cholerae* during the infection in *C. elegans*, VCC has been characterized as the toxin responsible for the cytopathic changes and lethality of the nematode. In response to the attack by VCC, *C. elelgans* activates the hypoxia pathway, and this pathway protects it against the PFT attack [26]. Hypoxia, a process of oxygen deprivation, has been implicated in various biological processes. VCC activates the hypoxia pathway resulting in the stabilization of the HIF-1 (Hypoxia Inducible Factor-1), and this stabilization in turn enhances the longevity of *C. elegans* [69]. Details of the pathway, and the mediators involved, have not been identified, but it is established that the stabilization of HIF-1 provides resistance to VCC, whereas loss of HIF-1 confers the nematode hypersensitive to the attack by VCC [26].

Apart from the responses mentioned above, attack by PFTs may initiate other responses in the host cells that increase the survival chances of the cells. Host cells may repair the damage by activating the sterol regulatory element binding protein (SREBP)-dependent pathway, which increases the expression of lipogenic genes that assist in repairing the pores formed in the plasma membranes by the PFTs [66]. SLO attack activates a repair mechanism that is a calcium/calmodulin-dependent resealing of the pores [80]. Therefore, it appears that the PFT-induced assault may evoke multiple possible cellular responses towards generating the survival strategies in the target cells. A few such responses have been documented in case of VCC. However, more detailed studies would be required to establish their physiological implications in the context of the VCC mode of action and *V. cholerae* pathogenesis process.

## 7. Conclusions

PFTs constitute an important class of bacterial toxins that play significant roles in the virulence mechanisms of a wide array of pathogenic bacteria. VCC, secreted by the pathogenic strains of *V. cholerae*, is a β-PFT that forms anion-selective pores in the target cell membrane, thus causing colloid-osmotic lysis of the cells.

**Figure 2:**
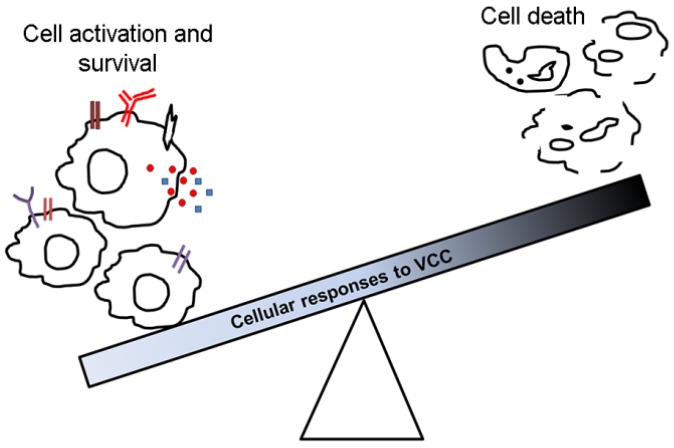
Two extreme scenarios of how a cell may respond to VCC. On one hand, cell death can be induced via membrane permeabilization, or through induction of apoptosis. On the other hand, activation of various protective mechanisms may provide the target host cells a chance to survive against the toxin attack. An intricate balance between these two responses may have critical implications in the context of the host–pathogen interaction processes during the *V. cholerae* infection.

Apart from the direct cell-killing mechanism via the membrane pore-formation process, VCC induces a plethora of cellular responses in the host cells. VCC triggers cytotoxicity by initiating caspase-dependent apoptosis and/or mechanisms like cell vacuolation. VCC is recognized mainly by TLR2 present on the cell surface that further instigates signaling cascades, activating the cells to respond to the toxin-induced assault. In response to attack by VCC, the host cells may produce inflammatory cytokines such as IL-8, IL-6 and TNFα. VCC punches a hole in the host plasma membrane, and to regain the cellular integrity, the host cells initiate autophagic pathway or enhance amounts of phosphorylated p38. Treatment of VCC to the *C. elegans* has been reported to activate hypoxia signaling that increase the longevity of the organism. In sum, VCC as a prototype bacterial PFT participates in the bacterial virulence processes, while the host cellular machinery can recognize VCC and elicit multiple responses that in turn may generate a protective mechanism against the toxin attack (Figure 2). Such a conflict during the cross-talk between the toxin and the target host cells is intriguing, and would have critical implications in the context of bacterial virulence mechanisms and the host-pathogen interaction processes.
